# Survival and Neurogenesis-Promoting Effects of the Co-Overexpression of *BCLXL* and *BDNF* Genes on Wharton’s Jelly-Derived Mesenchymal Stem Cells

**DOI:** 10.3390/life12091406

**Published:** 2022-09-09

**Authors:** Paulina Borkowska, Julia Morys, Aleksandra Zielinska, Marcin Sadlocha, Jan Kowalski

**Affiliations:** 1 Department of Medical Genetics, Medical University of Silesia, 41-200 Sosnowiec, Poland; 2 Chair and Department of Gynecology, Obstetrics and Gynecological Oncology, Medical University of Silesia, 40-211 Katowice, Poland

**Keywords:** mesenchymal stem cells, co-overexpression, BCLXL, BDNF, survival, neurogenesis

## Abstract

The main problem with using MSC (*mesenchymal stem cells*) to treat the deficient diseases of the central nervous system is the low cell survival rate after the transplant procedure and their low ability to spontaneously differentiate into functional neurons. The aim of this study was to investigate the effects of genetically modifying MSC. A co-overexpression of two genes was performed: *BCLXL* was supposed to increase the resistance of the cells to the toxic agents and *BDNF* was supposed to direct cells into the neuronal differentiation pathway. As a result, it was possible to obtain the functional overexpression of the *BCLXL* and *BDNF* genes. These cells had an increased resistance to apoptosis-inducing toxicants (staurosporine, doxorubicin and H_2_O_2_). At the same time, the genes of the neuronal pathway (*CHAT*, *TPH1*) were overexpressed. The genetically modified MSC increased the survival rate under toxic conditions, which increased the chance of surviving a transplant procedure. The obtained cells can be treated as neural cell progenitors, which makes them a universal material that can be used in various disease models. The production of neurotransmitters suggests that cells transplanted into the brain and subjected to the additional influence of the brain’s microenvironment, will be able to form synapses and become functional neurons.

## 1. Introduction

Mesenchymal stem cells seem to be an ideal candidate for use in cell-based therapy to treat many diseases, including neurodegenerative disease [1,2,3]. This is due to a number of their properties, such as their easy isolation from various types of tissue: Wharton’s Jelly [4,5], bone marrow [6], adipose tissue [7], and umbilical cord blood [8]; their ability to differentiate into cells from all three germ layers [9,10,11]; and their low tumorogenic potential. Due to the low level of MHC (*major histocompatibility complex*) protein expression, they can be used for allografts without the need for immunosuppression [12]. Although different groups of researchers have differentiated MSC into both dopaminergic [13,14,15] and cholinergic [16] neurons, using progenitor or differentiated neuron-like cells is quite difficult due to their high mortality rate after transplantation. The survival rate of grafted progenitors or neuroblasts is usually quite low, in the range of 5–20% for grafted fetal dopamine neurons [17] and a similar range for grafted hESC-derived (*human embryonic stem cell-derived*) dopamine neuron progenitors [18,19]. The addition of growth factors such as GDNF (*glial cell line-derived neurotrophic factor*) or cytoprotective agents (lazaroids, caspase inhibitors) can be used to increase this figure about 2-fold, a finding that has justified their use as additives in the cell preparations used in the clinical trials [17,20]. Both undifferentiated MSC and MSC differentiated into neural progenitors, in vitro are highly sensitive to the stroke transplant procedure. Additionally, it is not in the nature of MSC to spontaneously differentiate into neurons. As a result, MSC weakened by the transplant procedure die in the new and foreign brain microenvironment, which in some conditions is highly toxic for the transplanted material. In Alzheimer’s disease (AD), accumulation of beta amyloid triggers amyloidogenesis and hyperphosphorylation of tau protein leading to neuronal cell death [21]. Furthermore, free radicals present in the brain endogenously, produced cytokines [22] such as TNFα (*tumor necrosis factor α*) or IL-1β (*interleukin 1 beta*), irrespective of whether viruses [23] exacerbate the toxic properties of the brain’s environment. Therefore, the authors hypothesized that a solution to the above-mentioned problems could be a genetic modification of MSC that would make them resistant to toxic factors and at the same time directed to the neuronal differentiation pathway.

The principal objective of this examination was to overexpress two types of genes in the Wharton’s Jelly-derived MSC (WJ-MSC) for which one overexpressed gene, BCLXL (*B-cell lymphoma—extra-large*), is expected to enhance the resistance of cells to toxins and to increase their survival in an unfavorable environment and, thus, be anti-apoptotic [24,25], whereas the second, BDNF (*brain-derived neurotrophic factor*), is expected to enhance both neurogenesis [26] and synaptogenesis [27]. When the cells comprising a transplant integrate with the host tissue and perform their proper functions, there is a better chance they will survive [28]. The authors’ goal was to determine whether the overexpression of BDNF would modify the WJ-MSC in such a way that in the brain’s microenvironment, they would differentiate into nerve cell progenitors and then into specific neurons [29,30]. Thus, because of the increase in synaptogenesis due to the overexpression of BDNF [27], it would be possible to create a network of neurons, exhibiting specific conductance. 

During this experiment, we wanted to differentiate the WJ-MSC into neural cell progenitors. WJ-MSC that have differentiated into mature neurons are much more sensitive to stroke transplantation, which reduces the chance of transplant survival [31]. In addition, being able to obtain the progenitors in vitro will make the material for transplantation much more flexible. The obtained cells could then be used as material for transplantation for various diseases of the nervous system because the microenvironment of the brain largely determines which cells ultimately, in situ, differentiate into the neuronal progenitors [32]. The genetically modified WJ-MSC that are delivered to the brain might replace cells that have died, while also secreting neurotrophic and protective factors that would improve the functionality of the damaged area of the brain [29,30]. The success of the presented concept of modifying WJ-MSC will be a valuable clue for cell therapy for various tissue defects.

## 2. Materials and Methods

**Figure 1:**
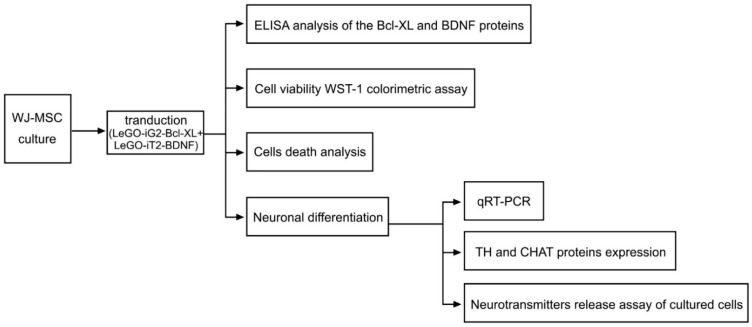
Overview of the general idea of the experiment. The diagram shows the experimental procedures that were performed on the FV group (Figure 2C+2D) in which there was a functional overexpression of the Bcl-XL and BDNF proteins. An analogous procedure was performed for the control group (C) and the EV group that had been transduced with empty vector backbones (Figure 2A+2B).

An overview of the general idea of this experiment is presented in Figure 1. We followed the methods of Borkowska et al. [4,33]. 

### 2.1. Isolating, Culturing and Characterizing the WJ-MSC

The WJ-MSC were isolated from human umbilical cords after delivery according to a previously published protocol [4]. The study was approved by the Bioethical Committee of the Medical University of Silesia in Katowice (Resolution No. KNW/0022/KB/195/14). The participants (mothers) were informed of the procedure in writing and gave their written consent to use the umbilical cords. The homogeneity of the WJ-MSC was quantified using flow cytometry; the presence of CD73, CD90, CD34, CD11b, CD19, CD45 and HLA-DR (*human leukocyte antigen–DR isotype*) was also determined. The WJ-MSC were characterized based on their differentiation capacity toward adipocyte and osteocyte cells [5]. All of the procedures for characterizing the WJ-MSC had previously been published [4].

### 2.2. Lentiviral Vectors

**Figure 2:**
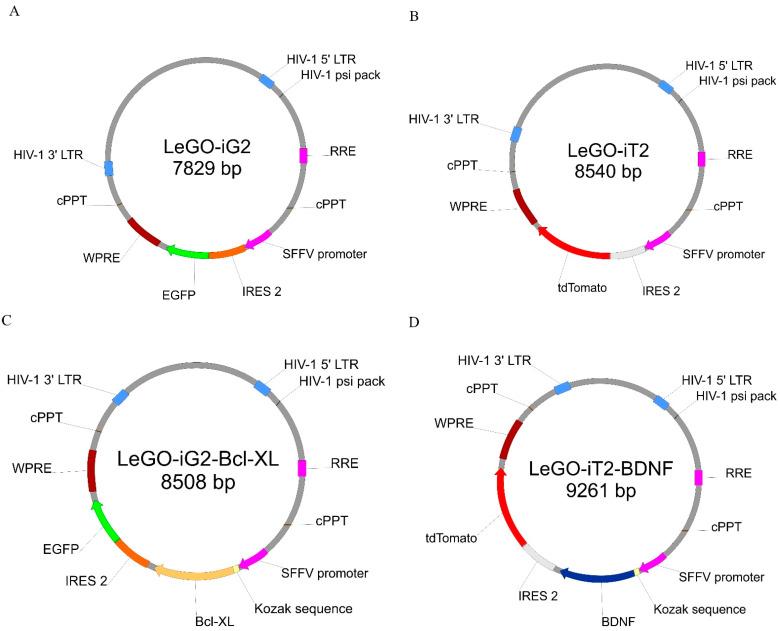
The simplified lentiviral vectors maps that were used for the overexpression. The sequence of the Bcl-XL gene enriched with the Kozak sequence was cloned into the plasmid (**A**) that contained the green fluorescence protein (EGFP) (**C**). The sequence of the BDNF gene enriched with the Kozak sequence was cloned into the plasmid (**B**) that contained the red fluorescence protein (tdTomato) (**D**). In the further parts of this work, plasmids A and B, which were used synergistically for the transduction, the abbreviations EV—empty vectors, and plasmids C and D under abbreviation FV—full vectors are used.

The lentiviral constructs were prepared in two different vectors so that the overexpression of the two genes would be constituted to the same degree. The plasmids used to produce the lentiviral vectors, the protein sequences that were to be overexpressed and the lentivirus production protocol had previously been published [4]. Because the coding sequences of *BCLXL* and *BDNF* were cloned into the empty LeGO-iG2 (Figure 2A) and LeGO-iT2 (Figure 2B) backbones, authors hoped for the obtained vectors- LeGO-iG2-Bcl-XL (Figure 2C) and LeGO-iT2-BDNF (Figure 2D) to allow the overproduction of the Bcl-XL and BDNF proteins in WJ-MSC.. Boris Fehse kindly provided the LeGO-iG2 (Addgene plasmid # 27341; http://n2t.net/addgene:27341; accessed on 20 November 2014; RRID:Addgene_27341) [33] and LeGO-iT2 (Addgene plasmid # 27343; http://n2t.net/addgene:27343; accessed on 20 November 2014; RRID:Addgene_27343) [34]. The vectors were designed in such a way that there was one vector in each pair into whose backbone the green fluorescence reporter gene (*EGFP*) was built. In this case, it was the LeGO-iG2-Bcl-XL vector. The second gene from each pair was built into the backbone with the red fluorescence reporter gene (*tdTomato*); in this case, it was the LeGO-iT2-BDNF vector. Additionally, in order to provide a functional increase in the overexpression that was generated and to increase the amount of proteins that were synthesized near the START codon of each gene, the Kozak sequence was added. The SFFV promoter controlled each of the vectors and lentiviral transduction was used to obtain the efficient and stable overexpression of the specific genes [35]. The same promoter was used to control the reporter genes as the one that was for the cloned sequences and the backbone was built so that the cloned gene was replicated first, which was then followed by the reporter gene. It was possible to determine the amount of the protein, produced on the gene sequence matrix that had been cloned into a given skeleton by analyzing the fluorescence intensity of the products of the protein reporter genes.

### 2.3. WJ-MSC Transduction, Protein Extraction

One day before they were transduced (200,000 cells per 35 mm dish Ø per culture), the WJ-MSC were plated in order to achieve a 70–80% confluence before the transduction. A 24 h incubation of the preconfluent cells with an optimal dilution of a virus in an Opti-MEM I Reduced Serum Medium (Life Technologies, Waltham, MA, USA; 31985070) in the presence of 5 μg/mL polybrene (without adding any antibiotics) was followed by a two-day culture of the cells in a normal medium DMEM/F12 (PAN Biotech, GmbH, Aidenbach, Germany; P04-41250) that had been supplemented with 15% FBS (PAN Biotech; P30-8500) and a 1% Antibiotic Antimycotic Solution (100X) (PAN Biotech; P06-07300) prior to the transduction. After two days of the culture, the medium was replaced with another one, showing a lower FBS content in order to inhibit cell proliferation (DMEM/F12 supplemented with 2% FBS and a 1% Antibiotic Antimycotic Solution (100X)), and the medium was then replaced twice a week. The cells were then cultivated for 60 days. Cells that had been transduced using empty vectors and a control culture to which no vectors were added at the transduction stage were cultured simultaneously. On days 3, 7, 14, 21 and 60 of the culture, the cells were lysed in order to determine the amount of Bcl-XL and BDNF protein production. After trypsinizing and pelleting at 1200 rpm for 5 min. at 4 °C, the cells were then washed once with PBS in a Ø 35 mm culture dish after which the cells were lysed in 200 µL of a RIPA buffer (10 mM Tris/HCl pH 7.4, 1 mM EDTA, 150 mM NaCl, 1% Nonidet P-40, 1% deoxycholate, 0.1% SDS, 1 mM PMSF), which had been supplemented with a 10% protease inhibitor cocktail (Sigma-Aldrich, Saint Louis, MO, USA; P8849-1ML). The pellets were pipetted on ice to homogenize them, and the obtained cellular material was pelleted by centrifuging at 13,000 rpm for 15 min. at 4 °C. The supernatant was portioned out and frozen at −80 °C until the samples from all of the time points had been collected.

### 2.4. ELISA Analysis of the Bcl-XL and BDNF Proteins

A commercially available ELISA human immunoassay kit: Human/Mouse Total Bcl-XL DuoSet IC ELISA (R&D Systems, McKinley Place, MN, USA; DYC894-2) and Human Free BDNF Quantikine ELISA (R&D Systems, DBD00) were used to measure the concentration of an overexpressed protein in the cells that had been cultured. An ELX 800 IU automated Microplate Reader (Bio-Tek Instruments, Inc., Winooski, VT, USA; Gene 5 Software V 3.02) was used to read the absorbance at 450 nm after which a quadratic log–log curve fit was used to analyze the results.

### 2.5. Cell Viability WST-1 Colorimetric Assay

The effect of the Bcl-XL and BDNF proteins on cell viability was determined using a WST-1 (4-(3-(4-iodophenyl)-2-(4-nitrophenyl)-2H-5-tetrazolio)-1,3-benzene disulfonate) (Roche Applied Science, Penzberg, Germany; 11644807001) colorimetric test. Roche’s WST-1 cell proliferation reagent was designed to measure the relative proliferation rates of cells in culture. The assay principle is based on the conversion of the tetrazolium salt WST-1 into a colored dye by mitochondrial dehydrogenase enzymes. Into the media, the soluble salt is released. Within a period of time, the reaction is shown as a color change, which is directly proportional to the amount of mitochondrial dehydrogenase in a given culture. As a result, the assay measures the net metabolic activity of cells, which is reflective of cell number—the more cells, the more dehydrogenase available to reduce the reagent. Due to this reason, the test may also be adopted for use in measuring cell viability or cytotoxicity. 

To perform the assay, 5000 cells were seeded into each well on 96-well plates. The method that was described above was used for the transduction after which the cells were then cultivated in a reduced FBS medium for 24 h or 14 days. Then, the concentration-dependent effect of staurosporine (0.5 and 1 μM) (Sigma-Aldrich; S4400), doxorubicin (1 μM and 5 μM) (Sigma-Aldrich; D1515), and hydrogen peroxide (1 and 1.5 mM) (Sigma-Aldrich; 216763) over a 24 h treatment was investigated. Moreover, the effect of a three-day deprivation of FBS was investigated.

After 24 h of treatment with an agent, a 100 µL 10% solution of WST-1 in DMEM/F12 without phenol red (PAN Biotech; P04-41650) was added to the culture medium, which was then incubated for 45 min at 37 °C. An ELX 800 IU automated Microplate Reader (Bio-Tek Instruments, Gene 5) was used to read the absorbance at 450 nm. Bio-Tek Gene 5 Software V 3.02 was used to analyze the results. The experiments were performed in four wells for each of the conditions and each was replicated at least three times in three independent experiments. To calculate the cell viability, the absorbance values of the samples were calculated after subtracting the background. The fraction of surviving cells was determined by defining the transduced cells vs. a negative control (WJ-MSC that had been cultured in the same conditions but that had not been transduced or treated with any agent).

### 2.6. Cell Death Analysis

For the assay, 50,000 cells were seeded into each well on 24-well plates. The cells were transduced as was described above, after which they were further cultivated in a lowered FBS medium for 14 days. Next, the resistance of the cells to 1 μM staurosporine for 12 h was determined and the manner of cell death was examined after it was applied. After trypsinizing and centrifuging (1200 rpm, 5 min, 4 °C), the cells were washed once in PBS. Next, Vybrant DyeCycle Violet/SYTOX AADvanced Apoptosis Kit (Life Technologies; A35135) were used to stain 1 × 10^6^ cells per mL, which makes it possible to distinguish normal, apoptotic and necrotic cell populations by the staining pattern that results from using both dyes simultaneously, as is described in the manufacturer’s protocol. Flow cytometry (BD FACSAria II; BD FACSDiva, San Jose, CA, USA, Software V6.1.2) was then used to analyze the results.

### 2.7. Neuronal Differentiation

In order to perform the assay, 50,000 cells were seeded into each well on 12-well plates. Poly-L-ornithine and fibronectin-coated vessels were used to perform all of the differentiation procedures. A differentiating medium was used to replace the transduction medium immediately after transduction. A medium containing Neurobasal PLUS (Gibco/Thermo Fisher Scientific, Waltham, MA, USA; A3582901) and B27 PLUS supplement (Gibco: A3582801) was used to induce neuronal differentiation. For the WJ-MSC cells that had been transduced by the “full vectors”, the neuronal differentiation medium was additionally supplemented with bFGF (5 ng/mL) (Gibco; PHG0264) or resveratrol (10 µM) (Sigma-Aldrich; 554325). The differentiation protocol was performed for 12 days. 

### 2.8. qRT-PCR

After the differentiation, the fresh cells were used to extract RNA. NucleoZOL (Macherey-Nagel, GmbH, Dueren, Germany; 740404.200) was used to extract the total cellular RNA according to the manufacturer’s protocol. Gel electrophoresis and spectrophotometry, respectively, were used to determine the quality and concentration of the RNA. The primers that were used for the selected genes, including *RPS17* (reference gene) (P_F_ 5′ CCATTATCCCCAGCAAAAAG 3′; P_R_ 5′ GAGACCTCAGGAACATAATTG 3′; Primer Pair ID H_RPS17_1), *SYP* (P_F_ 5′ CCCTTCGGTATTGTTCAAAG 3′; P_R_ 5′ TTTGACTAGGTGGTTAAGGAG 3′; Primer Pair ID H_SYP_1), *CHAT* (P_F_ 5′ TCATTTCTTTGTCTTGGATG 3′; P_R_ 5′ TGGAAGCCATTTTGACTATC 3′; Primer Pair ID H_CHAT_1), *TH* (P_F_ 5′ CAAAATCCACCATCTAGAGAC 3′; P_R_ 5′ CTGACACTTTTCTTGGGAAC 3′; Primer Pair ID H_TH_1) and *TPH1* (P_F_ 5′ AAAGAGCGTACAGGTTTTTC 3′; P_R_ 5′ GTCTCACATATTGAGTGCAG 3′; Primer Pair ID H_TPH1_1), were purchased from Sigma Aldrich (KiCqStart SYBR Green Primers). Amplicon sequencing in which a unique product is amplified based on electrophoresis in a 1–2% agarose gel was used to confirm the specificity of each primer pair and a melting-curve analysis was performed after the qRT-PCR assays were completed. A GoTaq 1-Step RT-qPCR System (Promega, GmbH, Walldorf, Germany; A6020) was used to perform the one-step qRT-PCR in triplicate. A 10 μL reaction with 15 ng of total RNA and a 0.2 μM final primer concentration for each forward and reverse primer in a C1000 Touch Thermal Cycler equipped with a CFX96 Real-Time System (Bio-Rad) was used to perform the qRT-PCR. The qRT-PCR procedure comprised a 15 min RT reaction at 37 °C, a 10 min PCR activation at 95 °C and then 40 cycles of a 10 s denaturation at 95 °C, a 30 s annealing at 60 °C at the lowest primer pair’s melting temperature and a 30 s PCR extension at 72 °C. Lastly, in order to confirm the qRT-PCR specificity, a melting-curve analysis was performed. Each run of the qRT-PCR included negative controls that had no total RNA. A Bio-Rad CFX96 Real-Time System (Bio-Rad CFX Manager; Marnes-la-Coquette, France, Software Version 3.1) was used to determine the Ct automatically.

### 2.9. Evaluating the TH and CHAT Expression of the Proteins

Indirect labeling with a specific anti-TH or anti-CHAT antibody was used to identify the TH or CHAT protein-positive cells. After the differentiation, the cells were washed twice in PBS and fixed in PBS with 4% paraformaldehyde (Sigma-Aldrich; 158127). The cells were then washed three times in PBS with 1% BSA (Sigma-Aldrich; A9418), permeabilized with 0.3% Triton X-100 (Sigma-Aldrich; T8787)/1% BSA/PBS with 5% normal goat serum (Jackson Immuno Research, Cambridge, UK; 005-000-121) for 45 min and incubated with rabbit anti-tyrosine hydroxylase antibody (dilution 1:600; AssayBioTech, Fremont, CA, USA; B0037) or with rabbit anti-choline acetyltransferase antibody (dilution 1:300; Proteintech, Manchester, UK; 20747-1-AP) in 1% BSA/PBS with 5% goat serum overnight at 4 °C. The cells were washed three times in PBS with 1% BSA and incubated with goat anti-rabbit IgG secondary antibody (DY405) (dilution 1:600; LSBio, Seattle, WA, USA; LS-C355899) in 1% BSA/PBS for 1 h in the dark at room temperature. In order to determine any nonspecific binding, a similar staining was conducted using a rabbit IgG isotype control (dilution 1:1500; Bioss Antibodies; bs-0295P). Flow cytometry was used to analyze the cells (BD FACSAria II; BD FACSDiva Software V6.1.2).

### 2.10. Neurotransmitters Release Assay of Cultured Cells

To perform the assay, 50.000 cells were seeded into each well on 12-well plates. After transduction, the cells were differentiated for a period of 12 days. The cells were then washed twice in PBS, after which the medium was replaced with a low K^+^ solution–Neurobasal-A (5.33 mM KCl) (Life Technologies; 10888022) for 30 min in a 250 µL/well. The low K^+^ medium was collected and 4 mM of Sodium Metabisulphite (Sigma-Aldrich; PHR1434) and 1 mM of EDTA (Sigma-Aldrich; E7889) was added. After the medium had been collected, the wells were refilled with a high K^+^ medium—Neurobasal-A medium, to which KCl had been added (Sigma-Aldrich; P9541); this was necessary in order to obtain a total of 53–56 mM KCl. Afterward, the cells were incubated for 30 min in a 250 µL high K^+^ solution/well. The high K^+^ medium was collected at the end of the termination period and 4 mM of Sodium Metabisulphite and 1 mM of EDTA were added to the medium. Both media (low and high K^+^) were then centrifuged at 13,000 rpm for 15 min. at 4 °C. The levels of the neurotransmitters (dopamine and acetylcholine) in the supernatant were determined immediately. The neurotransmitter levels were obtained and quantified using an ELISA (enzyme-linked immunosorbent assay) kit (Elabscience, E-EL-0046; E-EL-0081) according to the manufacturer’s instructions. An ELX 800 IU automated Microplate Reader (Bio-Tek Instruments; Gene 5 Software V 3.02) was used to read the absorbance at 450 nm. A quadratic log–log curve fit was used to analyze the results. The supernatants (low and high K^+^) were analyzed for both wells. In order to minimize the effect of the difference in the number of cells in the wells, the number of neurotransmitters that had been produced per well was calculated based on the difference: amount in high K^+^ supernatant–amount in a low K^+^ supernatant.

### 2.11. Statistical Analysis

The data are presented as the mean ± SD. The Shapiro–Wilk test for normal distribution was used for the analysis. A two-way ANOVA followed by a post hoc Tukey’s test was performed. GraphPad Prism (GraphPad Prism; Software version 8.0) was used to analyze the date. For all of the tests, *p* < 0.05 was considered to be statistically significant. The qRT-PCR results are presented as the fold change (2^−ΔΔCT^).

## 3. Results

**Figure 3:**
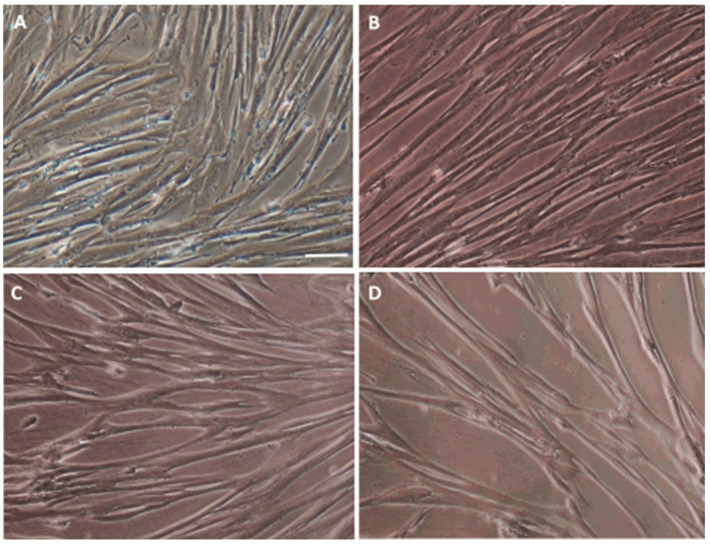
Characterization of an in vitro WJ-MSC culture. (**A**) Characteristic WJ-MSC fibroblast-like morphology. The WJ-MSC were cultured in a complete medium as is described in Materials and Methods. Changes in the cell morphology after a 12-day differentiation procedure in the control—C (**B**), empty vectors—EV (**C**) and full vectors—FV (**D**) groups. Scale bar = 50 µm.

The authors conducted the presented experiment in three main groups: (1) the control group abbreviated as C, in which the cells were subjected to procedures analogous to the other groups, but without the use of lentiviruses for the transduction or any stimulators in the differentiation process (Figure 3B); (2) the group that was synergistically transduced with the empty lentiviral backbones of the LeGO-iG2 (Figure 2A) and LeGO-iT2 (Figure 2B), which is abbreviated as EV (empty vectors), in which the differentiation was performed without any stimulators (Figure 3C); and (3) the test group that was synergistically transduced with a pair of lentiviral vectors LeGO-iG2-Bcl-XL (Figure 2C) and LeGO-iT2-BDNF (Figure 2D), which is abbreviated as FV (full vectors), in which the differentiation was carried out in various variants—without any stimulators, with the addition of bFGF (Figure 3D) or with the addition of resveratrol.

### 3.1. Characterization of the Isolated WJ-MSC

**Figure 4:**
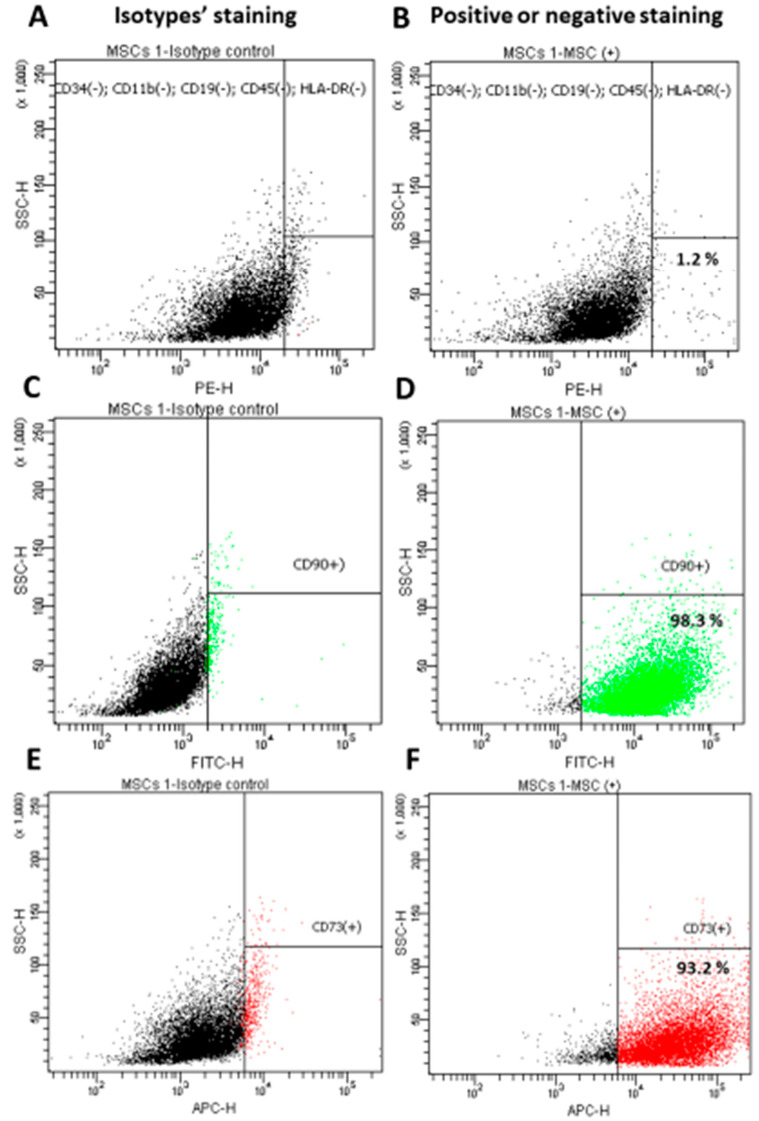
Immunophenotypic analysis for the WJ-MSC using flow cytometry. Isotype controls (**A**,**C**,**E**) were used for gating. Almost all of the WJ-MSC cells were negative for CD34, CD11b, CD19, CD45 and HLA-DR (**B**) and positive for CD90 (**D**) and CD73 (**F**). Data shown are representative of at least three independent experiments.

**Figure 5:**
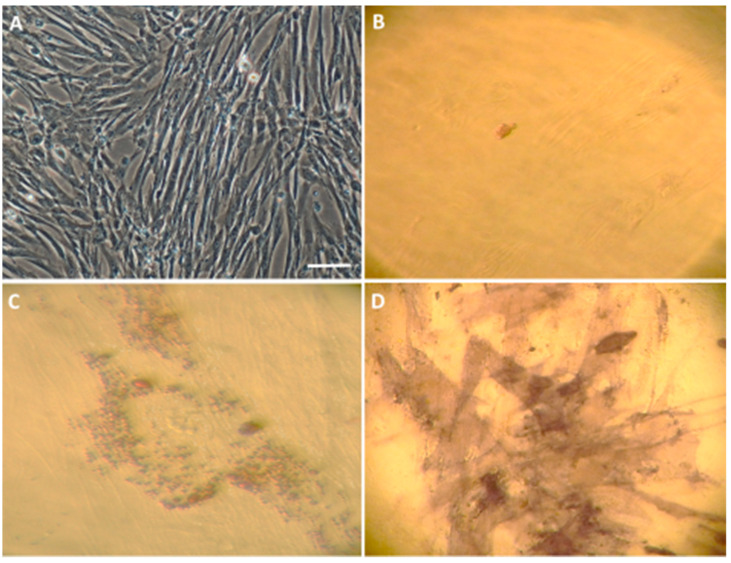
Characterization of an in vitro WJ-MSC culture. (**A**) Characteristic WJ-MSC fibroblast-like morphology. The WJ-MSC were cultured in a complete medium as described in Materials and Methods. (**B**) The control cultured in standard medium (DMEM/F12 with 0.5% FBS and a 1% Antibiotic Antimycotic Solution) equally with adipogenic and/or osteogenic differentiation (**C**,**D**). WJ-MSC were cultured for three weeks in either an adipogenic (**C**) or osteogenic (**D**) differentiation medium. Lipid vacuoles were stained with Oil Red O (**C**) and matrix mineralization was determined using alkaline phosphatase staining (**D**). Scale bar = 50 µm.

The first step in implementing the research objectives described above was isolating the Wharton’s Jelly-derived mesenchymal stem cells (WJ-MSC) (Figure 3A). For the purpose of the preliminary tests, WJ-MSC non-commercial, homogeneous lines were isolated, evaluated and banded. MSC are a heterogeneous cell population that is characterized by their spontaneous adherence to plastic; they have a typical immunophenotypic profile (expression of the surface markers CD73 and CD90, and lack of CD34, CD45, CD11b, CD19, CD45, and HLA-DR) (Figure 4) and a multilineage-differentiation potential into adipocytes and osteocytes (Figure 5).

### 3.2. Efficiency of WJ-MSC Transduction and Overexpressed Proteins Level

**Figure 6:**
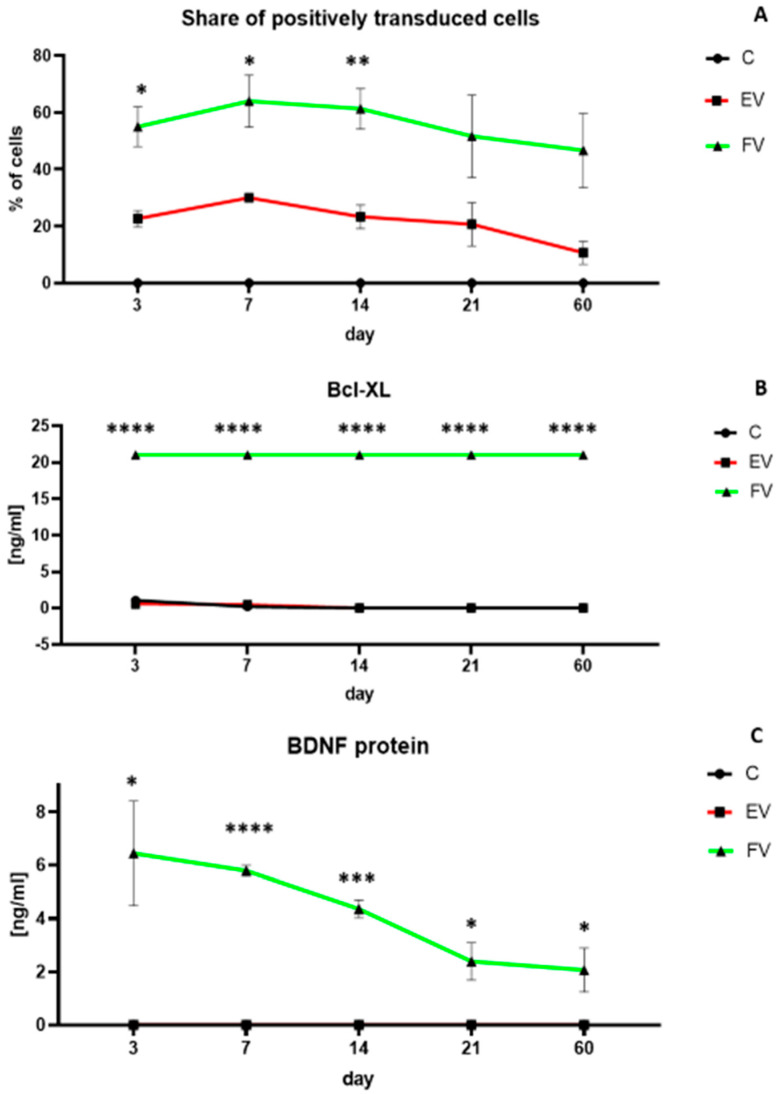
Percentage of positive transduced cells over time (**A**). Quantitative analysis of the Bcl-XL (**B**) and BDNF (**C**) protein production over time. At each time point, the experiment was performed for all three groups: cells overexpressing the genes *BCLXL* and *BDNF* (full vectors—FV); empty vector transduced cells—(EV); control (**C**). In (**A**), there were no positively transduced cells in C group. In (**C**), BDNF protein level of EV group is the same as the C group (0 ng/mL all the time). The points represent the mean value (n = 4; two independent experiments). Statistically significant * *p* < 0.05; ** *p* < 0.01; *** *p* < 0.001; **** *p* < 0.0001 two-way ANOVA test followed by a post hoc Tuckey’s test. Detailed statistical analysis is available in Supplementary Materials section.

The efficiency of the transduction process was monitored over time using the reporter proteins that were contained in the vectors. The overexpression with vectors from both the EV and FV groups was stable up to about 14 days after the moment of transduction, after which it decreased slightly, although even after 60 days, there was still a protein overexpression (Figure 6A). There was a clearly higher and statistically significant overproduction in both the Bcl-XL and BDNF proteins. Overproduction of the Bcl-XL protein was high and stable in the long-term culture even after 60 days (Figure 6B, EV vs. FV: *p* < 0.0001). A clear and statistically significant overproduction of the BDNF protein was observed up to about 14 days after transduction (Figure 6C, day 7 EV vs. FV: *p* < 0.0001; day 14 EV vs. FV: *p* < 0.001). After 14 days, BDNF overproduction decreased slightly, although even after 60 days there was still protein overexpression (Figure 6C, day 60 EV vs. FV: *p* < 0.05). Despite the standardization of viral titer, and application of the exact same titer of EV as well as FV, the transduction efficiency is higher in the second one. Similar technical inconveniences were observed in other studies. Given the significant diversity of multiple factors having an impact on the transduction process, we presume that the size of the used vector is meaningful in that case.

### 3.3. Cell Viability

**Figure 7:**
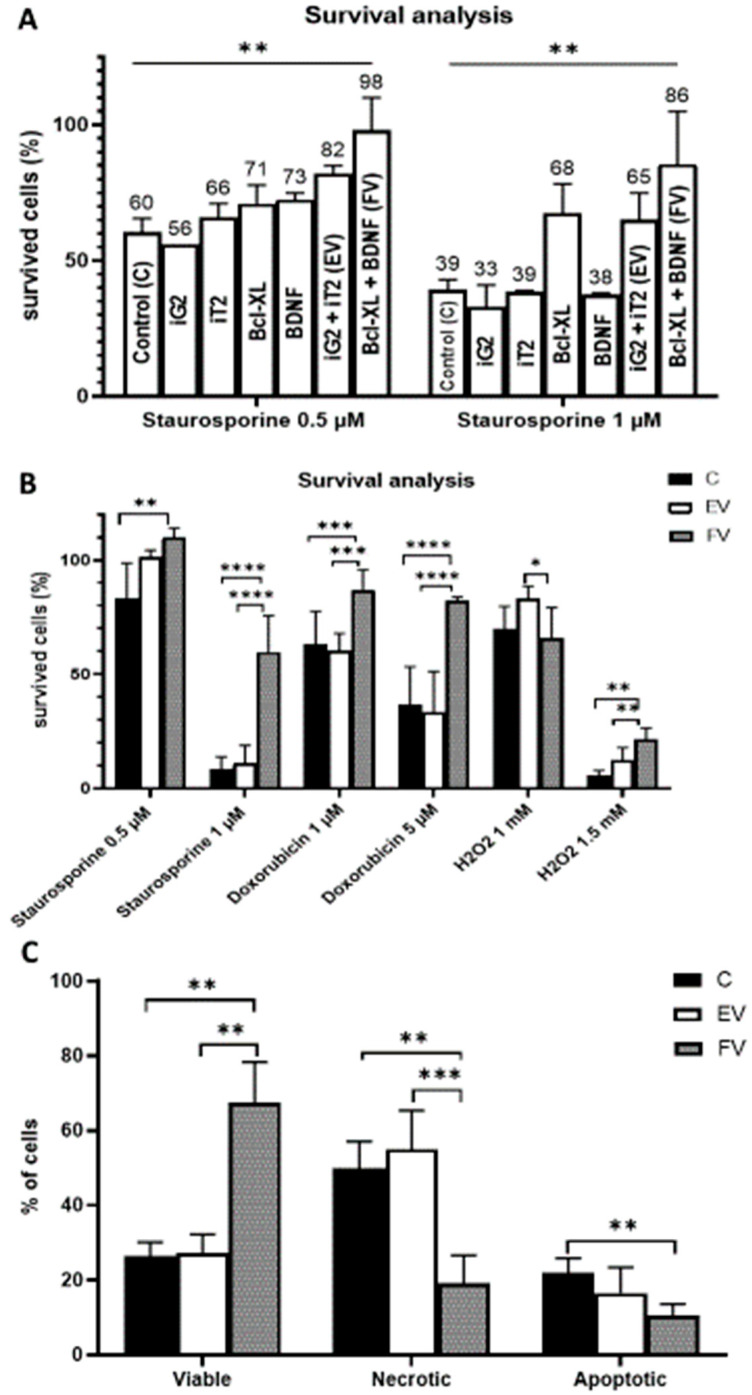
Cell survival analysis. Transduction with a single vector compared with a synergistic transduction of the empty vectors (EV: iG2 + iT2) or full vectors (FV: Bcl-XL + BDNF). The WST-1 was measured 24 h after the addition of staurosporine; two-way ANOVA test followed by a post hoc Tuckey’s test (**A**). Cell survival was analyzed after various death inducers were used. The WST-1 was measured 24 h after the addition of each agent; two-way ANOVA test followed by a post hoc Tuckey’s test. (**B**). An examination of cell resistance to a toxic agent and an analysis of its type of death. The measurement was conducted 12 h after the addition 1 µM of staurosporine; two-way ANOVA test followed by a post hoc Tuckey’s test. (**C**). Apoptosis induction was done one day after transduction ended for (**A**) and after 14 days for (**B**,**C**). The points represent the mean value ± SD (n = 12; three independent experiments). Statistically significant * *p* < 0.05; ** *p* < 0.01; *** *p* < 0.001 or **** *p* <0.0001.

To test the cytoprotective properties of the designed constructs, transduction using single constructs (Figure 2A–D) and synergistic pairs (Figure 2A+2B and 2C+D) was performed (Figure 7A). After the transduction had been performed, the cultures were grown for 24 h, and then the toxic agent staurosporine was applied in two doses (0.5 and 1 µM). After the application of 0.5 µM of staurosporine, the cytoprotective effect of the Bcl-XL overexpression was visible but, in this condition, BDNF also played a cytoprotective role (Figure 7A left, iG2 vs. Bcl-XL: ns; iT2 vs. BDNF: ns; Bcl-XL vs. FV: *p* < 0.001; BDNF vs. FV: *p* < 0.05). In much more toxic conditions, when 1 µM of staurosporine was used, the strong cytoprotective effect of Bcl-XL was confirmed (Figure 7A right, iG2 vs. Bcl-XL: *p* < 0.05; iT2 vs. BDNF: ns; Bcl-XL vs. FV: *p* < 0.001; BDNF vs. FV: ns). In addition, it was observed that the synergistic use of the vector pairs had an additive effect relative to their single application.

In order to test the stability of the cytoprotective properties of FV after transduction over time, the culture was grown for 14 days, i.e., until the transduction was relatively constant and the BDNF protein level was the highest. To confirm the cytoprotective properties of overexpression of two studied genes, cell death was induced by substances that are differing in the mechanism of action (Figure 7B). The cells that were overexpressing the Bcl-XL and BDNF proteins (FV) were significantly more resistant to staurosporine (1 µM), doxorubicin (1 and 5 µM) and H_2_O_2_ (1.5 mM). The protective role of the overexpressed proteins was particularly evident after high doses of the toxic agents were used: staurosporine 1 µM, doxorubicin 5 µM and H_2_O_2_ 1.5 mM. The more stressful the environment, the greater the protective potential for overexpression. Additionally, after a longer time from the performed transduction, the cytoprotective properties of the FV proteins were significantly enhanced (Figure 7B) compared to the test that had been performed immediately after transduction (Figure 7A).

### 3.4. Cell Death Analysis

In addition to demonstrating the cytoprotective properties of the overexpression of the Bcl-XL and BDNF (FV) proteins, an equally important aspect was to investigate the type of death of some of the cells that had died. After the chromatin condensation test was performed, it was shown that the cells overexpressing Bcl-XL and BDNF (FV) had a significantly higher survival rate (Figure 7C).

### 3.5. Neuronal Gene Expression Level

**Figure 8:**
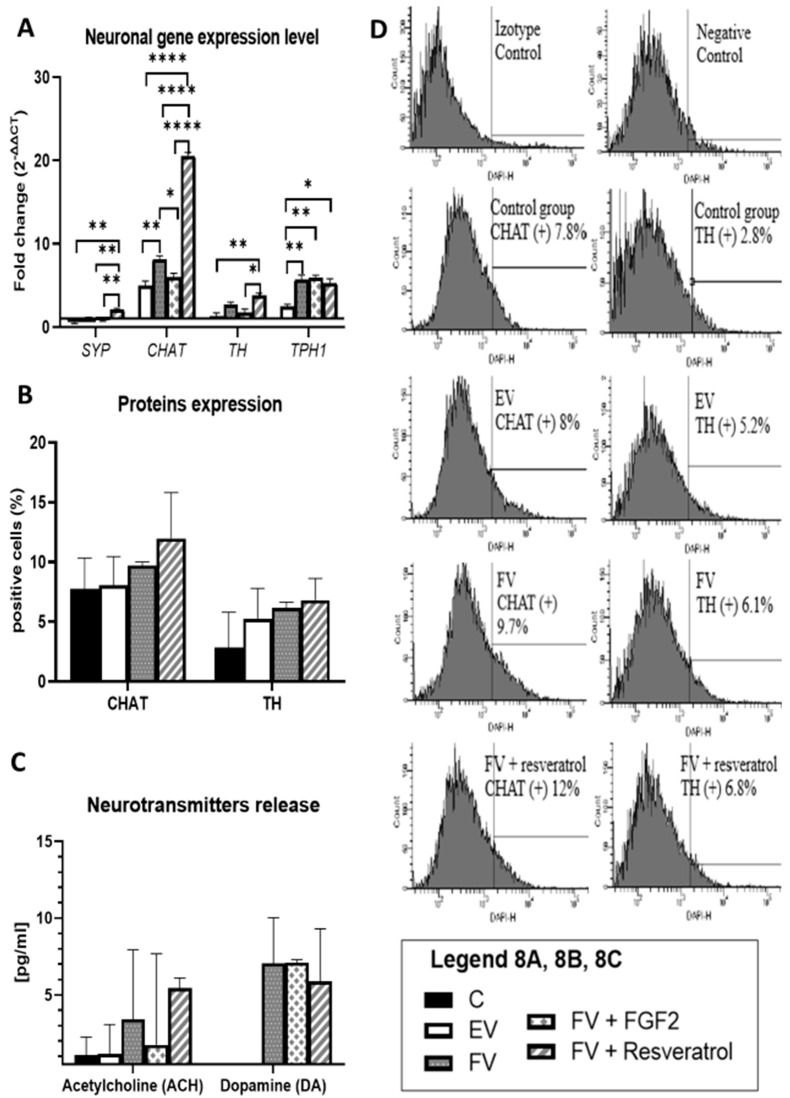
qRT-PCR analysis for the neuronal markers is presented as the fold change (2^−ΔΔCT^) in the level of their expression, which was normalized to the RPS17 reference gene; * *p* < 0.05, ** *p* < 0.01 or **** *p* < 0.0001 (**A**). The number of CHAT and TH positive cells. The calculations were based on a flow cytometry analysis. Points represent the mean value ± SD (n = 6; three independent experiments). Statistically significant * *p* < 0.05; two-way ANOVA test followed by a post hoc Tuckey’s test (**B**). Acetylcholine and dopamine release after depolarization by the neuronal-differentiated WJ-MSC. Points represent the mean value (n = 6; three independent experiments). Statistically significant * *p* < 0.05; two-way ANOVA test followed by a post hoc Tuckey’s test (**C**). Flow cytometric analysis for the expression of neural specific proteins: CHAT (choline acetyltransferase) and TH (tyrosine hydroxylase) after 12 days of differentiation. Percentage values represent the mean value (n = 6; three independent experiments) (**D**). Control group (**C**), the group that had been transduced with empty vectors (EV), the group of cells that had an overproduction of the Bcl-XL and BDNF proteins (FV) and in FV that had additionally been supplemented with bFGF or resveratrol.

In the presented experiment, after the differentiation procedure, the expression level of the synaptophysin (*SYP*) gene, which is one of many genes that is responsible for the synaptogenesis process, and the genes of the neuronal pathway, *CHAT* (cholinergic neurons), *TH* (dopaminergic neurons) and *TPH1* (serotoninergic neurons), were investigated. It can be concluded that after the overexpression of the Bcl-XL and BDNF (FV) genes, there was an increase in the expression level of *CHAT* and *TPH1* genes (Figure 8A, EV vs. FV: *p* < 0.01). Due to this, the resulting cells can be thought of as a heterogeneous population of neuronal progenitors rather than as a homogeneous population of neurons of a specific type.

### 3.6. TH and CHAT Protein Expression

The increased level of gene expression may not translate directly into the expression level of characteristic proteins of the neuron-like cells, that is why no statistically significant results were observed. In order to confirm this, CHAT (choline acetyltransferase)–cholinergic neurons) and TH (tyrosine hydroxylase)–dopaminergic neurons proteins were analyzed. In the group in which the Bcl-XL and BDNF (FV) genes were overexpressed and in the FV + resveratrol group, although statistically insignificant, the level of the expression of the proteins that are typical of neurons was higher (Figure 8B,D).

### 3.7. Neurotransmitter Release

The most important feature that cells must have is their functionality. In the case of neuron-like cells, this is assessed on the basis of the ability to produce neurotransmitters. It was shown that cells in which the Bcl-XL and BDNF (FV) genes were overexpressed had the ability to produce increased amounts of acetylcholine and dopamine. Similar results were obtained when a resveratrol supplement was used in the differentiation procedure. Conversely, the addition of bFGF during differentiation in a targeted manner increased the ability of cells to produce dopamine (Figure 8C). Furthermore, what should be mentioned is that the absence of any statistically significant results in this part does not mean that no significant change occurred. Neurotransmitters are substances that are released in such a low amount that any slight increase or decrease might show a therapeutic effect. That being said, even though there was a lack of significance statistically, the amount of neurotransmitters might still be meaningful for therapeutic reasons.

## 4. Discussion

The principal objective of this research was the evaluation of effects observed in MSC that underwent genetic modification. As a result of taken measures, involving the co-overexpression of *BDNF* as well as *BCLXL*, modified cells exhibited an increased resistance to apoptosis-inducing toxicants, such as staurosporine. Moreover, the second feature achieved was the overexpression of neural pathway genes (*CHAT*, *TH*). Bearing in mind that WJ-MSC are a promising material to use in regenerative medicine, especially in the treatment of neurodegenerative disorders, obtained properties seem to be significantly valuable. One such disorder is Parkinson’s disease (PD), which is characterized by the selective loss of the dopamine (DA) neurons in the human midbrain [36]; however, aside from PD, it is difficult to find other neurodegenerative diseases that are associated with one type of neuronal loss in one area of the brain. For these reasons, we did, as well as the other authors, decide to use MSC, which have a natural ability to migrate to damaged areas, as the material for this research [37,38]. Furthermore, WJ-MSC was selected for research among other types of MSC because of the relative ease of obtaining. The umbilical cord is a material that is disposed of after delivery, so its use for further research does not raise ethical questions. In addition, the method of isolating MSC from Wharton’s jelly is easy and efficient. The ideal situation would, therefore, be one in which genetically modified and pre-differentiated WJ-MSC for nerve cell progenitors could be a relatively universal material for transplantation in the future. When they are injected into the olfactory bulb or injected intracerebroventricularly (i.c.v.) [39], they migrate to damaged sites and, because they are modulated by the microenvironment in a given region of the brain, they differentiate into the neurons of a specific phenotype [29,30]. Modified WJ-MSC that is transplanted into the brain in diseases such as PD, AD and Huntington’s chorea (HD) should differentiate in the microenvironment of the brain tissue and integrate with the neurons that live in the brain. The last finding shows that transplanted MSC-FGF21 are able to migrate successfully toward the injured hemisphere [40]. Furthermore, transplantation of these MSC-FGF21 cells to the brain of mice after traumatic brain injury effectively reduced brain lesion volume, improved learning/memory, normalized dendritic morphology and stimulated neurogenesis in the dentate gurus [40]. Despite the numerous advantages of WJ-MSC, the main problem with using them is their relatively low survival rate after the stroke transplant procedure and their low tendency to spontaneously differentiate into different neurons.

In order to solve the above-mentioned problems, the concept of performing the overexpression of the two genes *BCLXL* and *BDNF* has emerged. One gene in the pair (*BCLXL*) is designed to make the cells stronger and therefore to increase their viability after the transplant procedure is performed, while the other (*BDNF*) is designed to guide the cells into the differentiation pathway of the neurons. 

The very concept of examining the properties of the overexpression of these two genes is innovative and has not yet been studied. In addition, the stability of the overexpressed genes was studied for the first time. It was determined in our study, that transduction remains stable over time and that even as many as 60 days after the procedure was performed, the number of cells that had been transduced was significantly higher compared to the cells in the control and empty vector groups. The effectiveness of transduction was confirmed by the expression of the *EGFP* protein. The significant overproduction of Bcl-XL was observed up to the 60th day of culture, and, simultaneously, we observed that the cytoprotective effect of the Bcl-XL protein appears. The stability of BDNF gene overexpression translated into the stability of the BDNF protein overproduction and has also been observed by other researchers [41,42]. The long-term overproduction of proteins is extremely important for testing the genetically modified cells that were obtained in future studies of neurodegenerative diseases in animal models. The transduction must be stable long enough after a transplant to give the cells time to adapt to their new environment and ultimately to differentiate into a specific cell type, which can integrate with the host tissue and then perform their proper functions.

The protective effect of the overexpression of single genes was then compared to that of the overexpression of two genes. The anti-apoptotic role of Bcl-XL is well known [24,25,43] and was confirmed in this experiment. The overexpression of the *BCLXL* gene increased the cell survival rate by 100% compared to the control group. BDNF is an important signaling molecule that contributes to the maintenance of the functional integrity and survival of neurons that are under stress [44,45]. However, in the present experiment, there was no improvement in the cytoprotective properties after the overexpression of BDNF. On the other hand, there was an additive effect of the *BCLXL* and *BDNF* genes that had been overexpressed to increase the ability of the cells to survive under toxic conditions. 

Following the other researchers [46], to confirm the cytoprotective properties of overexpression of two studied genes, cell death was induced by substances which are differing in the mechanism of action: staurosporine, doxorubicin and H_2_O_2_. The mechanism by which staurosporine induces death is not clear. A previous experiment showed that staurosporine can induce apoptosis and/or necroptosis in cultured cells via different signaling pathways [47]. Apoptosis is induced through both caspase-dependent and caspase-independent mechanisms [48] but necroptosis is induced by caspase-independent mechanisms [49]. Doxorubicin induces cell death by autophagy, ferroptosis, necroptosis, pyroptosis and apoptosis. Final mechanisms of action depend on culture conditions [50]. The association of ferroptosis with the pathophysiology of some neurodegenerative diseases, namely, AD, PD, and HD, has also been addressed [51]. H_2_O_2_ is well known as a factor, which causes oxidative cell death by ROS (reactive oxygen species) generating, which causes damage to DNA and other macromolecules [52]. Because oxidative stress plays a major role in AD, there is also significant evidence of enhanced oxidative stress in PD because the markers of oxidative damage to biological structures, such as the oxidation of lipids, proteins, and DNA are higher in PD [53]. Due to the fact that the previous experiment confirmed that staurosporine exposure is useful as a model for studying central neuronal apoptosis in vitro [54,55,56], the authors evaluated resistance to the toxic conditions with staurosporine. The experiment showed that the more toxic the conditions, the better the protective role of the overexpressed proteins [57,58,59]. The toxic conditions that are generated in vitro might be a reflection of the stroke transplant procedure. Thus, demonstrating the increased viability of genetically modified cells after the application of, e.g., 1 µM of staurosporine, might indicate their increased ability to survive the stroke transplant procedure. In addition to the procedure itself, the conditions in the recipient’s brain may also be neurotoxic. In AD, the accumulation of beta amyloid triggers amyloidogenesis and the hyperphosphorylation of the tau protein, which leads to neuronal cell death [21]. In HD, the accumulation of β-catenin in its phosphorylated form was reported, and reduction in β-catenin levels inhibits the toxic effects of mutant huntingtin in vitro and in vivo [60]. In addition, some evidence supporting a role for oxidative damage in the etiology of neuronal damage and degeneration in HD [61]. Moreover, some toxins of natural origin: cycad-derived toxins, epoxomicin, *Nocardia asteroides* bacteria, *Streptomyces venezuelae* bacteria, annonacin and DOPAL possibly represent a contributory environmental component to PD [62,63]. Furthermore, the free radicals that are present in the brain endogenously produce cytokines [22] such as TNFα or IL-1β, whereas viruses [23] exacerbate the neurotoxic properties of the brain’s environment. 

An examination of the expression level of the genes and proteins that were generated on their matrix showed that a gene overexpression guided the WJ-MSC into the neuronal differentiation path. None of the studied directions of differentiation can be considered to be preferential. The expression of genes showed that after differentiation, the typical genes of the cholinergic, dopaminergic and serotonergic neurons were overexpressed, and also that the proteins that are typical of the cholinergic or dopaminergic neurons had similar percentages. Many data have indicated that Bcl-XL plays a major role in modulating the generation of the dopaminergic neurons from both rodent- and human-derived stem cells [64,65]. A previous study contributed data that indicated a more general role of Bcl-XL, i.e., controlling the balance between the generation of neurons and glia from differentiating immortalized and non-immortalized hNSCs [66]. BDNF is known to be a factor that promotes the survival of the GABAergic neurons [59]. BDNF has also been described as a factor which, due to its ability to suppress the death receptors on the surface of dopaminergic neurons, determines their increased survival, which in turn slows down the development of PD [67]. The influence of the overexpressed *BCLXL* and *BDNF* genes on the ability of MSC to differentiate into cholinergic or serotonergic neurons has not yet been studied. In one study, it was shown that BDNF, when used together with bFGF, enabled the differentiation of MSC of various origins into cells from the neuronal lineage; however, similar to this study, the authors obtained a mixture of the dopaminergic, cholinergic and serotonergic neurons with a two-fold quantitative predominance of the dopaminergic neurons [14]. Though the BDNF overexpression is expected to enhance neurogenesis, which was previously proved by other researchers [26], in our research there was no such phenomenon. It might be due to the different experimental model, based on the co-overexpression of the BCLXL and BDNF genes. Thus, it is not possible to directly compare it to the conclusions drawn by other authors by whom the co-overexpression of two genes has not been studied.

Depolarization made it possible to confirm that the obtained cells were capable of producing neurotransmitters. At the level of the study of the gene or protein expression, no preferential differentiation was observed after the overexpression of Bcl-XL and BDNF was used. Only after examining the ability to produce neurotransmitters was it observed that the amount of dopamine that was produced was significantly greater than the amount of acetylcholine that was produced. The works of other authors also confirmed the electrophysiological activity of the obtained cells and/or their ability to produce neurotransmitters [13,14,15]. BDNF is known to increase the amount of dopamine that is produced in the hippocampus [68] and the acetylcholine that is produced in the brain [69]. It has also been shown that an increase in the serum BDNF concentration is associated with an increased concentration of the serum neurotransmitters including dopamine [70]. 

It is possible to guide cells into the neural differentiation pathway due to the overexpression of Bcl-XL/BDNF The protocol proposed as a result of these studies can be further investigated in in vivo experiments on neurodegenerative diseases in various animal models because, in addition to the gene overexpression, the ultimate role that determines the direction of differentiation is played by the brain’s microenvironment.

## 5. Conclusions

The above-described experiment showed that:

1. The overexpressed *BCLXL* and *BDNF* genes increase the survival rate of the cells that have been transduced under toxic conditions, which increases their chance of survival after a stroke transplant procedure.
2. The overexpressed *BCLXL* and *BDNF* genes guide the transduced cells into the neuronal differentiation pathway. As a result, nerve cell progenitors can be obtained. These cells can therefore be a universal material that can be used in studies of various disease models.
3. The cells that are obtained as a result of the process produce neurotransmitters, which might suggest that after the cells have been transplanted into the brain, and are then subjected to the additional impact of the brain’s microenvironment, they might have a valuable impact on further therapeutic measures.

## Data Availability

The datasets used and/or analyzed during the current study are available from the corresponding author on reasonable request.

## Supplementary Materials

The following supporting information can be downloaded at: https://www.mdpi.com/article/10.3390/life12091406/s1, Detailed statistical analysis.
