# Supplementary material for: Survival and Neurogenesis-Promoting Effects of the Co-Overexpression of BCLXL and BDNF Genes on Wharton’s Jelly-Derived Mesenchymal Stem Cells
Source: Life (Basel). 2022 Sep 9;12(9):1406. doi: 10.3390/life12091406 (PMC9501059; doi:10.3390/life12091406)
Supplement: Supplementary file 1 [file life-12-01406-s001.zip › life-1863246-supplementary.pdf]

Detailed statistical analysis to Figure 6.

BDNF					
**ANOVA table**	**SS**	**DF**	**MS**	**F (DFn, DFd)**	**P value**
Time x Vector	41,27	8	5,159	F (8, 36) = 21,04	P<0,0001
Time (3/7/14/27/60)	20,64	4	5,159	F (1,254, 11,29) = 21,04	P=0,0004
Vector (C/EV/FV)	236,5	2	118,3	F (2, 9) = 160,7	P<0,0001

Post-hoc BDNF analysis (p value)					
	**t=3**	**t=7**	**t=14**	**t=27**	**t=60**
**C vs EV**	X	X	X	X	X
**C vs FV**	0,0145	<0,0001	0,0002	0,0133	0,0304
**EV vs FV**	0,0145	<0,0001	0,0002	0,0133	0,0304

BCL-XL					
**ANOVA table**	**SS**	**DF**	**MS**	**F (DFn, DFd)**	**P value**
Time x Vector	1,942	8	0,2428	F (8, 36) = 4,100	P=0,0015
Time (3/7/14/27/60)	2,914	4	0,7285	F (1,082, 9,736) = 12,30	P=0,0053
Vector (C/EV/FV)	5749	2	2875	F (2, 9) = 19663	P<0,0001

Post-hoc BCL-XL analysis (p value)					
	**t=3**	**t=7**	**t=14**	**t=27**	**t=60**
**C vs EV**	0,6073	0,7242	X	X	X
**C vs FV**	<0,0001	<0,0001	<0,0001	<0,0001	<0,0001
**EV vs FV**	<0,0001	<0,0001	<0,0001	<0,0001	<0,0001

**ANOVA table**	**P value**	**P value summary**	**F (DFn, DFd)**
Time (3/7/14/27/60)	0,0540	ns	F (1,314, 7,558) = 4,862
Vector (C/EV/FV)	<0,0001	****	F (2, 6) = 137,1
Time x Vector	0,2383	ns	F (8, 23) = 1,427

Post-hoc Share of positively transduced cells analysis (p value)					
	**t=3**	**t=7**	**t=14**	**t=27**	**t=60**
**C vs EV**	0,0098	0,0265	0,0190	0,0768	0,0804
**C vs FV**	0,0098	0,0123	0,0081	0,0458	0,0455
**EV vs FV**	0,0150	0,0377	0,0065	0,0907	0,0604
